# Induction of protein catabolism in myotubes by 15(*S*)-hydroxyeicosatetraenoic acid through increased expression of the ubiquitin–proteasome pathway

**DOI:** 10.1038/sj.bjc.6601184

**Published:** 2003-08-12

**Authors:** A S Whitehouse, J Khal, M J Tisdale

**Affiliations:** 1 Pharmaceutical Sciences Research Institute, Aston University, Birmingham B4 7ET, UK

**Keywords:** 15(*S*)-hydroxyeicosatetraenoic acid (15-HETE), protein catabolism, protenscore induction, nuclear factor-*κ*B (NF-*κ*B)

## Abstract

The potential role of 15(*S*)-hydroxyeicosatetraenoic acid (15(*S*)-HETE) as an intracellular signal for increased protein catabolism and induction of the expression of key components of the ubiquitin–proteasome proteolytic pathway induced by a tumour cachectic factor, proteolysis-inducing factor has been studied in murine C_2_C_12_ myotubes. 15(*S*)-HETE induced protein degradation in these cells with a maximal effect at concentrations between 78 and 312 nM. The effect was attenuated by the polyunsaturated fatty acid, eicosapentaenoic acid (EPA). There was an increase in ‘chymotrypsin-like’ enzyme activity, the predominant proteolytic activity of the proteasome, in the same concentration range as that inducing total protein degradation, and this effect was also attenuated by EPA. 15(*S*)-hydroxyeicosatetraenoic acid also increased maximal expression of mRNA for proteasome subunits C2 and C5, as well as the ubiquitin-conjugating enzyme, E2_14k_, after 4 h incubation, as determined by quantitative competitive RT–PCR. The concentrations of 15-HETE affecting gene expression were the same as those inducing protein degradation. Western blotting of cellular supernatants of myotubes treated with 15(*S*)-HETE for 24 h showed increased expression of p42, an ATPase subunit of the regulatory complex at similar concentrations, as well as a decrease in expression of myosin in the same concentration range. 15(*S*)-hydroxyeicosatetraenoic acid activated binding of nuclear factor-*κ*B (NF-*κ*B) in the myotube nucleus and stimulated degradation of I-*κ*B*α*. The effect on the NF-*κ*B/I-*κ*B*α* system was attenuated by EPA. In addition, the NF-*κ*B inhibitor peptide SN50 attenuated the increased chymotrypsin-like enzyme activity in the presence of 15(*S*)-HETE. These results suggest that 15(*S*)-HETE induces degradation of myofibrillar proteins in differentiated myotubes through an induction of an increased expression of the regulatory components of the ubiquitin–proteasome proteolytic pathway possibly through the intervention of the nuclear transcription factor NF-*κ*B, and that this process is inhibited by EPA.

Proteolysis through the ubiquitin–proteasome pathway has an important role in the regulation of the cell cycle and division, differentiation and development, as well as a variety of other basic cellular processes (Ciechanover *et al*, 2000). In addition, this pathway plays an important role in the wasting of skeletal muscle seen in a range of catabolic processes, including starvation, sepsis, metabolic acidosis, weightlessness, severe trauma, denervation atrophy and cancer cachexia (Lecker *et al*, 1999). In this process, proteins are degraded into peptides within the 20S proteasome, a cylindrical structure of four stacked rings, each composed of seven subunits surrounding a central cavity. The inner *β* rings contain proteolytic enzymes, while the two outer *α* rings surround a small cavity through which protein substrates must enter. Cellular proteins are marked for degradation by attachment of a polyubiquitin chain. While the ubiquitin-conjugating enzyme (E2_14k_) was suggested to be the rate-limiting step in the pathway in starvation (Wing and Banville, 1994), it has recently been shown that E2_14k_ knockout is not associated with decreased protein catabolism (Adegoke *et al*, 2002). Instead, ubiquitin-protein ligases (E3) may play a more important role in muscle atrophy and E3 knockout is clearly associated with increased protein catabolism (Bodine *et al*, 2001; Gomes *et al*, 2001).

Despite the importance of the ubiquitin–proteasome pathway, there is little information on intracellular signals leading to an increased gene transcription of the rate-limiting enzymes. It has been suggested that glucocorticoids and a low insulin level in tandem activate the ubiquitin–proteasome proteolytic system (Mitch *et al*, 1999). Glucocorticoids increase protein catabolism by opposing the suppression of the transcription of the proteasome C3 *α*-subunit by nuclear factor-*κ*B (NF-*κ*B), by antagonising the interaction of NF-*κ*B with an NF-*κ*B response element in the C3 subunit promoter region (Du *et al*, 2000). Also physiological levels of glucocorticoids are necessary, but not sufficient for the catabolic response in rats with metabolic acidosis (Price *et al*, 1994), starvation (Wing and Goldberg, 1993) and sepsis (Tiao *et al*, 1996). However, chronic excessive glucocorticoid production, as occurs in Cushing's syndrome is not associated with an increased mRNA for ubiquitin, the ubiquitin-conjugating enzyme, E2_14k_, or proteasome subunits (Ralliere *et al*, 1997), suggesting that other factors may be involved.

We have isolated a sulphated glycoprotein of Mr 24 000 produced by cachexia-inducing murine and human tumours (Todorov *et al*, 1996), which induces proteolysis directly in isolated skeletal muscle (Lorite *et al*, 1997) and in C_2_C_12_ murine myoblasts in the absence of glucocorticoids (Smith *et al*, 1999). This factor, named proteolysis-inducing factor (PIF), induces release of arachidonic acid from muscle cells with an increased production of prostaglandin (PG) E_2_ and F_2*α*_ together with metabolism through the lipoxygenase pathways to 5-, 12-, and 15-hydroxyeicosatetraenoic acids (HETEs) (Smith *et al*, 1999). Release of arachidonic acid and its subsequent metabolism was shown to be directly related to the induction of protein catabolism, since it was completely attenuated in the presence of eicosapentaenoic acid (EPA), which also completely inhibited protein degradation induced by PIF. Of the metabolites of arachidonate only 15(*S*)-HETE produced a significant increase in protein degradation alone, suggesting that it acts as an intracellular signal for the action of PIF. Since PIF has been shown to upregulate the ubiquitin–proteasome pathway (Lorite *et al*, 1998), this suggests that 15(*S*)-HETE should also induce the key regulatory components of this pathway.

This study utilizes an *in vitro* model of skeletal muscle (C_2_C_12_ myotubes) to investigate the effect of 15(*S*)-HETE on protein degradation and protein and mRNA levels of the key enzymes of the ubiquitin–proteasome pathway, as well as the involvement of potential transcription factors.

## MATERIALS AND METHODS

### Materials

L-[2,6-^3^H]Phenylalanine (sp. act. 2.00 TBq mmol^−1^) was purchased from Amersham International (Bucks, UK). Foetal calf serum (FCS), horse serum (HS) and Dulbecco's modified Eagle's medium (DMEM) were purchased from Life Technologies (Paisley, Scotland). Mouse anti-p42 antibody was a gift from Dr Jane Arnold, UK. Mouse monoclonal antibody to myosin heavy chain was purchased from Novocastra (Newcastle-upon-Tyne, UK). Rabbit polyclonal antisera to residues 1–317 of human I-*κ*B*α* was purchased from Santa Cruz Biotechnology Inc., CA, USA. Peroxidase-conjugated rabbit antimouse antibody was purchased from Dako Ltd, Cambridge, UK and peroxidase-conjugated goat anti-rabbit antibody from Sigma Chemical Co., Dorset, UK. 15(*S*)-HETE was purchased from Biomol Research Laboratories Inc., PA, USA. Oligonucleotides for EMSA, T4 polynucleotide kinase 10 × buffer and gel shift binding buffer were from Santa Cruz Biotechnology Inc., CA, USA. SN50 was purchased from Calbiochem, Nottingham, UK. Wizard™ mini or maxi preps, used to prepare plasmid DNA from overnight cultures of bacterial clones, were obtained from Promega, UK (Southampton), as were the T7 RNA polymerase kit, RNAsin inhibitor, reverse transcriptase, reverse transcription buffer and PCR grade magnesium chloride. Taq polymerase and Hybond C were from Amersham Biosciences, UK Ltd (Bucks, UK), TRI reagent for RNA isolation was purchased from Sigma-Aldrich, Co-Ltd (Dorset, UK), while the oligonucleotide primers were from MWG Biotech (Ebersberg, Germany). RNA storage buffer was purchased from Ambion Ltd (Cambridgeshire, UK) and PCR buffer from Roche, Switzerland.

### Cell culture

The C_2_C_12_ myoblast cell line was grown in DMEM supplemented with 10% FCS plus 1% penicillin and streptomycin under an atmosphere of 10% CO_2_ in air at 37°C. Myotubes were formed by allowing confluent cultures of myoblasts to fuse in DMEM containing 2% HS over a 7-day period, with changes of medium every 2 days.

### Measurement of total protein degradation

This was measured essentially as previously described for murine myoblasts (Lorite *et al*, 2001). Myotubes were formed in six-well multidishes containing 2 ml DMEM and 2% HS. Cells were labelled for 24 h with L-[2,6-^3^H] phenylalanine (0.67 mCi mmole^−1^), and washed three times in PBS. They were then incubated for 2 h in DMEM without phenol red until no more radioactivity appeared in the supernatant, and then for a further 24 h period with various concentrations of 15(*S*)-HETE in fresh DMEM without phenol red to reduce quenching of counts, and in the presence of 2 mM cold phenylalanine to prevent reincorporation of radioactivity. The amount of radioactivity released into the medium was determined using a 2000CA Tri-Carb liquid scintillation analyzer and is expressed as a percentage of control cultures determined from myotubes incubated with the [^3^H]phenylalanine for 24 h, but not treated.

### Measurement of proteasome activity

The functional activity of the *β*-subunits of the proteasome was determined as the ‘chymotrypsin-like’ enzyme activity determined fluorimetrically according to the method of Orino *et al*, (1991). Cells were sonicated in 20 mM Tris-HCl, pH 7.5, 2 mM ATP, 5 mM MgCl_2_ and 1 mM dithiothreitol (DTT) at 4°C and enzyme activity was determined on the supernatant fraction, formed by centrifugation for 10 min at 18 000 **g**, using the fluorogenic substrate succinyl-LLVY-AMC (0.1 mM) in the presence or absence of 10 *μ*M lactacystin, a specific proteasome inhibitor (Fenting and Schreiber 1998). Only lactacystin suppressible activity was considered to be proteasome specific. The reaction was terminated by the addition of 80 mM sodium acetate, pH 4.3, and the fluorescence determined with an excitation wavelength of 360 nm and an emission wavelength of 460 nm. The activity was adjusted for the protein concentration of the sample, determined using the Bradford assay (Sigma Chemical Co., Dorset, UK). Both substrate and enzyme concentrations were optimal and the results were similar to those obtained using purified proteasomes (Lorite *et al*, 2001).

### Western blot analysis

Myotubes were treated with various concentrations of 15(*S*)-HETE, with or without 2 h preincubation with 50 *μ*M EPA. The medium was removed and the cells were washed with PBS, followed by scraping from the plastic surface and sonication in 500–2000 *μ*l of the buffer used to measure proteasome activity. After centrifugation (18 000 **g** for 5 min), the supernatant was assayed for protein concentration using a colourimetric protein assay (Biorad, UK). Samples of cytosolic protein (2.5 *μ*g) were resolved on 12% sodium dodecylsulphate, polyacrylamide gels (SDS/PAGE) and transferred to 0.45 *μ*m nitrocellulose membranes (Hybond A, Amersham, UK), which had been blocked with 5% Marvel in Tris-buffered saline, pH 7.5, at 4°C overnight. The primary antibodies were used at a 1 : 1500 dilution, except for myosin heavy chain used at 1 : 250 dilution and I-*κ*B*α* at 1 : 400. The secondary antibodies were used at a 1 : 2000 dilution. Incubation was for 1 h at room temperature and development was by enhanced chemiluminescence (ECL) (Amersham, UK). Blots were scanned by a densitometer to quantitate differences, and a parallel gel was silver stained to ensure equal loading.

### Quantitative competitive RT–PCR

Total RNA was extracted from muscle using TRI-reagent and the RNA concentration was determined from the absorbance at 260 nm. To quantitate the mRNA of interest, increasing amounts of competitor RNA, which differed from the mRNA of interest by containing a short deletion, were added to 250 ng of total RNA and coamplified using RT–PCR. The amount of specific mRNA was calculated from the amount of competitor, when equal amounts of PCR product were obtained from the competitor and target RNA, as previously described (Wang *et al*, 1989; Taylor *et al*, 1998).

The C2 competitor was obtained from the mouse gene using the primer pairs: 5′CGCACGGAGTGCTGGTTGCAC3′ and 5′GTACGA GCTGATTGAGAACGG–CATAACCAGCAATGAGCA GCC3′ at positions 176–187 and 531–552 bp to 435–455 bp, respectively, which produced a DNA fragment containing a 76-bp deletion. The C5 competitor was obtained from the mouse gene using the primer pairs: 5′TCAACGGAGGTACTGTATTGG3′ and 5′GCATGGCACTT GCTGAGCC–GCATGGCACTTGCTGAGCC3′ at positions 101–121 and 496 to 514 bp to 358–378 bp, respectively, producing a DNA fragment containing a 117-bp deletion. The E2_14k_ competitor was obtained from the rat gene using the primer pairs: 5′CTCATGC GGGATTTCAAGCG3′ and 5′CTCTTCTCATACTCCCGTTTGCAT–C GGTTCTGCAGGATGTC3′ at positions 68–87 and 443–468 bp to 312–321 bp, respectively, producing a DNA fragment containing a 111-bp deletion. The competitor DNAs were blunt ended and then ligated into a pET30a vector, which had been blunt-end cut with *Sma*l restriction enzyme. The ligated vector was used to transform DH5*α* competent cells. Plasmid DNA was prepared from PCR-positive clones using Wizard-mini-prep. Competitor RNA was produced using T7 RNA polymerase kit, and quantitated using the optical density at 260 nm. Six serial two-fold dilutions were prepared containing known concentrations of competitor. The particular dilutions used were selected to span the selected concentration of C2, C5 and E2_14k_ in the sample.

To synthesise the cDNA template, a mixture consisting of 250 ng target RNA, the particular dilution of the competitor RNA and 0.5 *μ*g of random hexamer was incubated at 70°C for 5 min in a thermal cycler (Genetic Research, Instrumentation Ltd, Essex, UK) and then chilled on ice before the addition of 2.5 *μ*l 5 × reverse transcription buffer, 3 *μ*l of 10 mM each at dATP, dGTP, dCTP, dTTP, 5 U of RNAsin inhibitor and 1 U reverse transcriptase in a total volume of 12.5 *μ*l. Incubation was at 37°C for 1 h. For the amplification of the cDNA by PCR, 50 *μ*l of PCR mix was added to each tube. The PCR mixture contained 1 × PCR buffer (without magnesium) together with 3 mM MgC1_2_ (E2), 3.5 mM MgCl_2_ (C5) and 2.5 mM MgCl_2_ (C2) together with 1 U Taq polymerase. For the E2 gene, 10 pmol of each of the primers 5′CTCATGCGGGA TTTCAAGCG3′ and 5′CTCTTCTCATACTCCCGTTTG3′ were used, while for the C5 gene 10 pmol of each of the primers 5′TCAAC GGAGGTACTGTATTGG3′ and 5′GCATGGCACTTGCTGAGCC 3′ were used. For the C2 gene, 20 pmol of each of the primers 5′CGCA CGCAGTGCTGGTTGCAC3′ and 5′GTACGAGCTGATTGAGAAC GG3′ were used. The temperature-cycling profile for amplification was as follows: 95°C for 2 min for one cycle followed by 95°C for 30 s, 58°C annealing for 1 min and 72°C extension for 1 min for 30 cycles. Control reactions containing all components except reverse transcriptase and another without template were carried out alongside each experiment to show that the RNA (both target and competitor) had no DNA contamination, while the second control showed that there was no contamination in the PCR mixture. Coamplification of E2 target and competitor produced DNA fragments of 395 and 284 bp, respectively; C2 target and competitor produced 385 and 309 bp fragments and C5 target and competitor produced 414 and 297 bp fragments, respectively.

**Figure 1:**
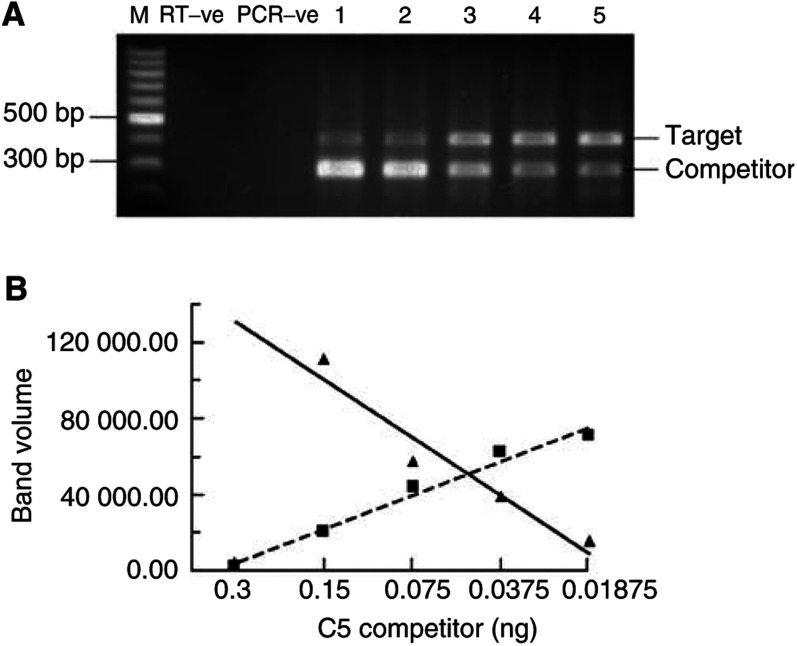
(**A**) Representative gel illustrating quantitative competitive RT–PCR for C5 mRNA in control cultures at 4 h incubation. Lanes: **M**; 100-bp DNA ladder: RT-ve; without reverse transcriptase, PCR-ve: without template. Lanes 1–5 each contain 0.25 *μ*g target RNA and a specific amount of the C5 competitor RNA ranging from 0.01875 to 0.3 ng in two-fold dilutions. (**B**) Dose–response curve from the gel shown in (**A**). ▪, target volume; ▴, competitor volume. The amount of unknown determined from where the two lines intersect=0.0445 ng mRNA.

For analysis of results, 15 *μ*l of PCR products were separated on a 2% (w v^−1^) agarose gel containing ethidium bromide. The gel images were visualised on a UV transilluminator and photographed. The intensity of the bands were quantitated using a Phoretix photo-imager programme. The volumes of the competitor and target bands were plotted against the known serial dilutions of the competitor used in the experiment. The amount of the sample RNA corresponds to the amount of competitor when the ratio of competitor to target is 1.0. A representative gel showing competitor and target RNA, as well as a dose–response curve is shown in Figure 1

.

### Preparation of nuclear proteins

Cell monolayers were rinsed, scraped and pelleted in 10 mM HEPES, pH 7.5, 10 mM KCl, 2 mM MgCl_2_, 1 mM DTT, 0.1 mM EDTA, 0.4 mM PMSF, 0.2 mM NaF, 0.2 mM sodium orthovanadate and 0.3 mg ml^−1^ leupeptin. The pellets were resuspended in 300 *μ*l of the same wash buffer and incubated on ice for 15 min. Lysis was achieved by the addition of 30 *μ*l of 1% Triton X-100, followed by vortexing, and nuclei were pelleted by a 30 s centrifugation at 14 000 r.p.m., and the supernatant was thoroughly removed. The nuclear pellet was resuspended in 50 *μ*l ice-cold lysis buffer (50 mM HEPES, pH 7.8, 50 mM KCl, 0.3 M NaCl, 0.1 mM EDTA and 1 mM DTT) and the suspension was kept on ice for 20 min with a 30 s vortex every 3–5 min. Centrifugation at 14 000 r.p.m. for 5 min yielded the supernatant containing the protein extract.

### Electrophoretic mobility shift assay

The transcription factor consensus oligonucleotides for the NF-*K*B-responsive element (5′AGT TGA GGG GAC TTT CCC AGG C3′) was purchased from Promega (Southampton, UK). The probes were labelled with [*γ*-^32^P]ATP by using T4 polynucleotide kinase (Promega) for 10 min at 37°C. For the binding reaction, nuclear extract (10 *μ*g protein) was incubated in a 10 *μ*l volume with 2 *μ*l EMSA binding buffer (20% glycerol; 5 mM MgCl_2_; 250 mM NaCl; 2.5 mM EDTA; 2.5 mM DTT; 50 mM Tris-HCl, pH 7.5 and 0.25 mg ml^−1^ poly (dI-dC)-poly (dI-dC) and 1 *μ*l *γ*-^32^P-labelled probe for 20 min at room temperature following a 10 min preincubation in the absence of the labelled probe. The specificity of the binding reaction was determined by coincubating duplicate samples with unlabelled probes. A negative control reaction was also included which contained everything except sample. Protein–nucleic acid complexes were resolved using an 8% nondenaturing polyacrylamide gel and a native 5% polyacrylamide stacking gel in 25 mM Tris, pH glycine for 30 min at 150 mV. Gels were dried between Whatman 3 M filter paper (Whatman Inc., Clifton, NJ, USA), dried under vacuum at 80°C for 1 h and exposed to ‘Hyperfilm MP’ (Amersham Pharmacia Biotech) for 48 h at −70°C.

### Statistical analysis

Results are presented as mean±s.e.m. Differences were determined by one-way ANOVA, followed by Tukey's test.

## RESULTS

**Figure 2:**
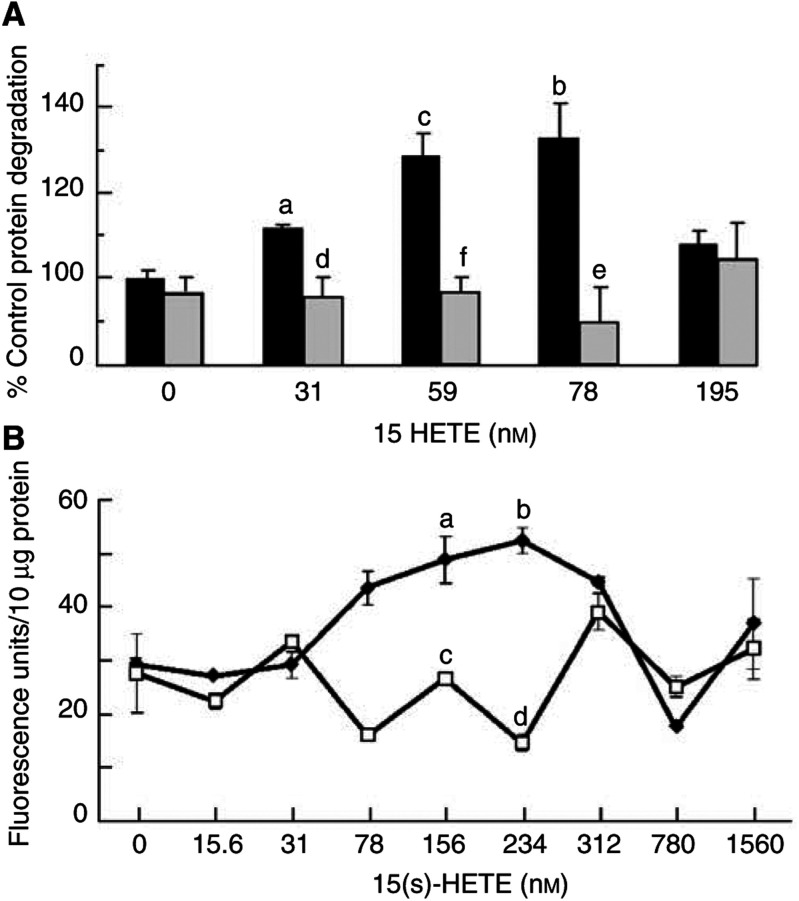
(**A**) Effect of 15(*S*)-HETE on total protein degradation in C_2_C_12_ myotubes after incubation for 24 h in cells either olive oil-treated (closed boxes) or pretreated for 2 h with 50 *μ*M EPA (hatched boxes). Results are shown as mean±s.e.m. of one experiment where *n*=3, and the experiment was repeated three times on different days with similar results. Differences from controls in the absence of 15(*S*)-HETE are indicated as (a) *P*<0.05, (b) *P*<0.01 or (c) *P*<0.001, while differences in the presence of EPA from those in the absence are indicated as (d) *P*<0.05, (e) *P*<0.01 and (f) *P*<0.001. (**B**) The effect of 15(*S*)-HETE on the proteasome ‘chymotrypsin-like’ enzyme activity in C_2_C_12_ myotubes either alone (⧫) or after pretreatment with 50 *μ*M EPA (□). Results are shown as mean±s.e.m. where *n*=3 and the experiment was repeated three times with similar results. Differences from controls in the absence of 15(*S*)-HETE are indicated as (a) *P*<0.05 and (b) *P*<0.01, while differences in the presence of EPA are indicated as (c) *P*<0.01 and (d) *P*<0.001.

The effect of 15(*S*)-HETE on total protein degradation in C_2_C_12_ myotubes, as measured by [^3^H]phenylalanine release, is shown in Figure 2A

. The concentrations of 15(*S*)-HETE chosen were based on those produced by PIF in murine myoblasts (Smith *et al*, 1999). In this study PIF increased 15-HETE production from 25 to 70 nmol per 5 × 10^6^ cells per 2 h, that is, an increase of 9 nmol per 10^6^ cells. If all the 15-HETE entered the cells in the current experiments, this would give a value between 1.4 and 6 nmol per 10^6^ cells. As previously reported in C_2_C_12_ myoblasts, 15(*S*)-HETE produced a significant increase in [^3^H]phenylalanine release with a bell-shaped dose–response curve, although the concentrations producing maximal protein catabolism (78–156 nM) were higher than required in myoblasts (Smith *et al*, 1999). A similar dose–response curve is observed with PIF (Smith *et al*, 1999), and with protein breakdown in muscle of tumour-bearing animals (DeBlaauw *et al*, 1997), and may result from downregulation of receptors, or receptor desensitisation as occurs in the stimulation of lipolysis by isoprenaline. The effect was attenuated down to control values in the presence of 50 *μ*M EPA at all concentrations of 15(*S*)-HETE (Figure 2A), as well as the highly specific and irreversible proteasome inhibitor, lactacystin (data not shown), suggesting that the action of 15(*S*)-HETE may be mediated through the ubiquitin–proteasome proteolytic pathway. This concentration of EPA has been previously shown to attenuate PIF-induced protein catabolism in murine myoblasts (Smith *et al*, 1999). Lower concentrations of EPA had no effect. The plasma concentration of EPA in humans consuming large doses would be in the range 100–200 *μ*M. To determine if proteasome proteolytic activity was also increased by 15(*S*)-HETE, the ‘chymotrypsin-like’ enzyme activity, the predominant proteolytic activity of the proteasome, was determined using the fluorogenic substrate Suc LLVY-MCA. The results presented in Figure 2B show that 15(*S*)-HETE induced upregulation of the ‘chymotrypsin-like’ activity of the proteasome, with maximal effect in the concentration range 78–312 nM, as for total protein catabolism (Figure 2A). As with protein catabolism, proteasome proteolytic activity was attenuated in cells pretreated with 50 *μ*M EPA.

**Figure 3:**
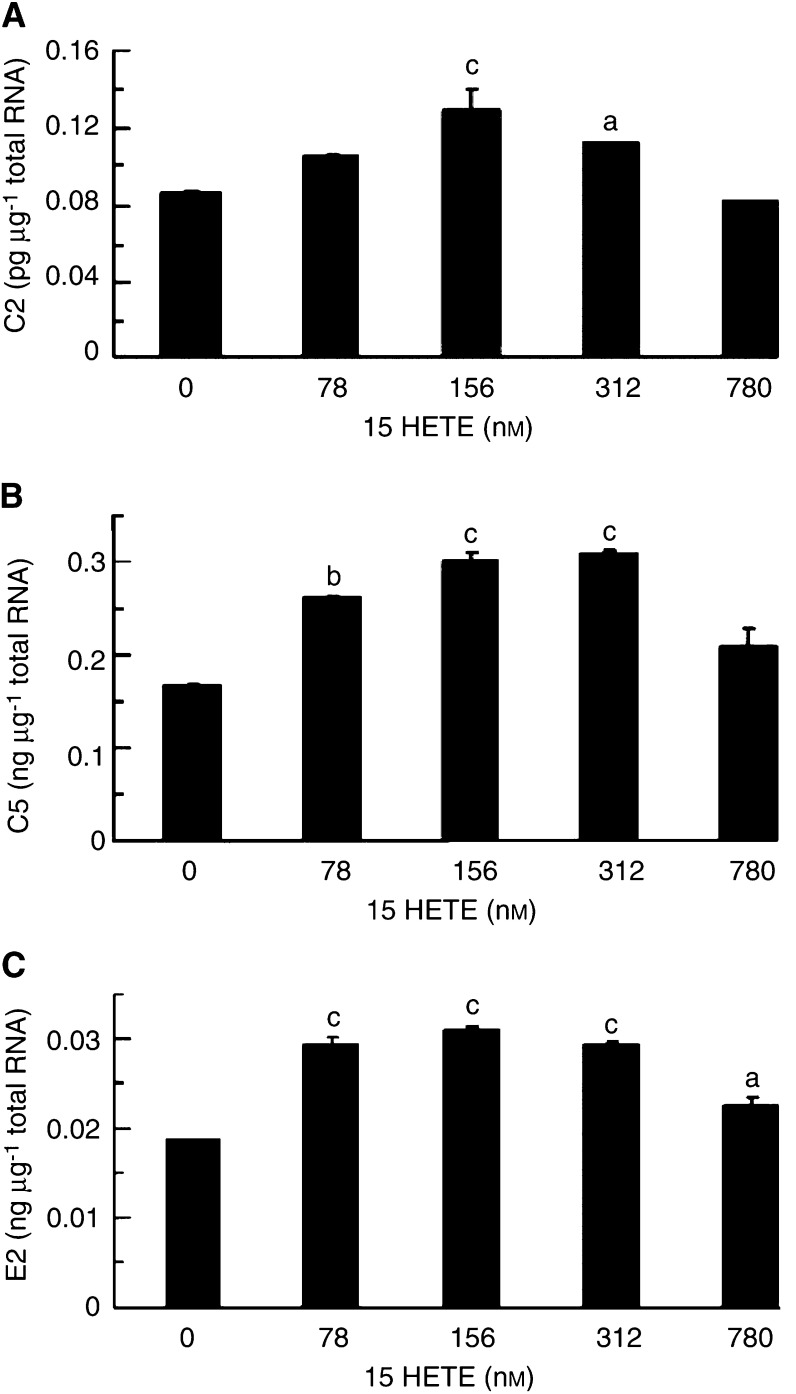
Effect of treatment of C_2_C_12_ myotubes with 15(*S*)-HETE for 4 h on expression of mRNA for (**A**) C2 proteasome subunit, (**B**) C5 proteasome subunit, (**C**) E2_14k_, as determined by quantitative competitive RT–PCR. Results represent mean±s.e.m. from three separate determinations performed on different occasions with different samples. Differences are indicated as (a) *P*<0.05, (b) *P*<0.01 and (c) *P*<0.001 from cells treated with 0 nM 15(*S*)-HETE.

**Figure 4:**
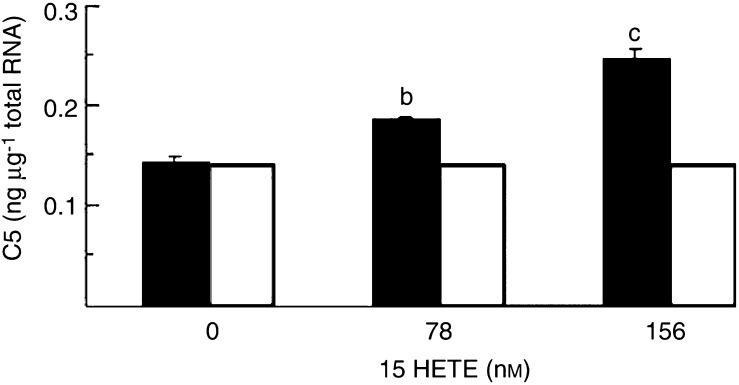
Effect of 15(*S*)-HETE on the expression of mRNA for the C5 proteasome subunit in C_2_C_12_ myotubes in the absence (solid boxes) and presence (open boxes) of 50 *μ*M EPA. Results represent mean±s.e.m. from two separate determinations performed on different occasions with different samples. Where there was no differences between individual measurements no standard error is shown. Differences from control are indicated as (b) *P*<0.01 and (c) *P*<0.001 as determined by Student's *t*-test.

**Figure 5:**
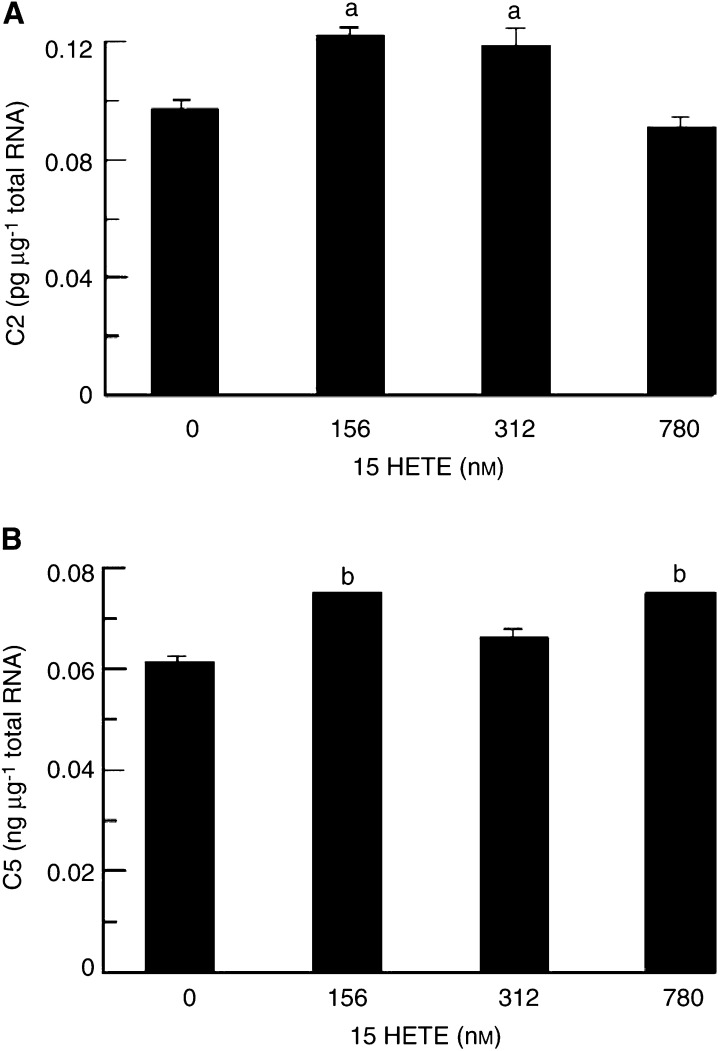
Effect of treatment of C_2_C_12_ myotubes with 15(*S*)-HETE for 2 h on expression of mRNA for (**A**) C2 proteasome subunit, (**B**) C5 proteasome subunit. Results represent mean±s.e.m. for three separate determinations on different samples. Differences from control are indicated as (a) *P*<0.05 and (b) *P*<0.001.

**Figure 6:**
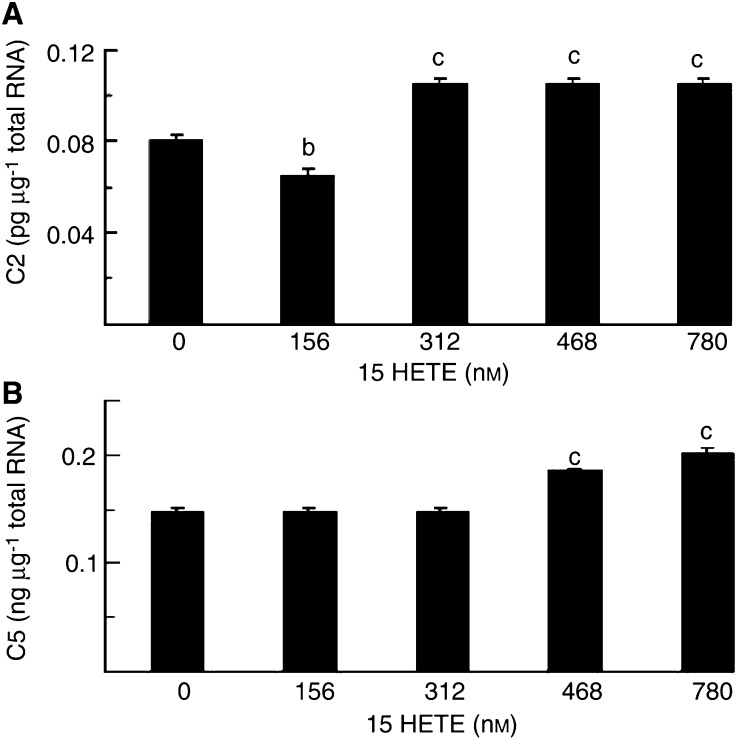
Effect of treatment of C_2_C_12_ myotubes with 15(*S*)-HETE for 24 h on expression of mRNA for (**A**) C2 proteasome subunit, (**B**) C5 proteasome subunit, as determined by quantitative competitive RT–PCR. Results represent mean±s.e.m., where *n*=3 representing three separate determinations on different samples. Differences from control are indicated as (b) *P*<0.01 and (c) *P*<0.001.

To determine whether the increased proteasome proteolytic activity arose from an increased gene expression mRNA levels of proteasome subunits C2(*α*) and C5(*β*), as well as the ubiquitin-conjugating enzyme, E2_14k_, were determined in C_2_C_12_ myotubes exposed to various concentrations of 15(*S*)-HETE for various times to determine the earliest time of gene induction and how long this persisted, using quantitative competitive RT–PCR (Figure 3

). Expression of the mRNA for the 20S proteasome subunits C2 (Figure 3A) and C5 (Figure 3B) was significantly increased with 15(*S*)-HETE concentrations in the range 78–312 nM after 4 h, as for protein catabolism with a similar bell-shaped dose–response curve. The level of expression of mRNA for C5 was approximately 2000-fold greater than for C2. Expression of mRNA for E2_14k_ was also significantly increased 4 h after treatment with 15(*S*)-HETE (Figure 3C), although the concentration range was somewhat broader than for proteasome subunits (78–780 nM). The extent of induction of C2, C5 and E2_14k_ by 15(*S*)-HETE at 2 h (Figure 5

) was less than that observed at 4 h. The increased expression of proteasome subunits in the presence of 15(*S*)-HETE was attenuated by prior treatment with EPA (Figure 4

). There was also evidence for an increased expression of mRNA for both C2 (Figure 6A

) and C5 (Figure 6B) proteasome subunits after 24 h. As with studies on PIF (Gomes-Marcondes *et al*, 2002), higher concentrations of 15(*S*)-HETE produced increases in proteasome mRNA expression at 24 h than at 4 h (Figure 3).

**Figure 7:**
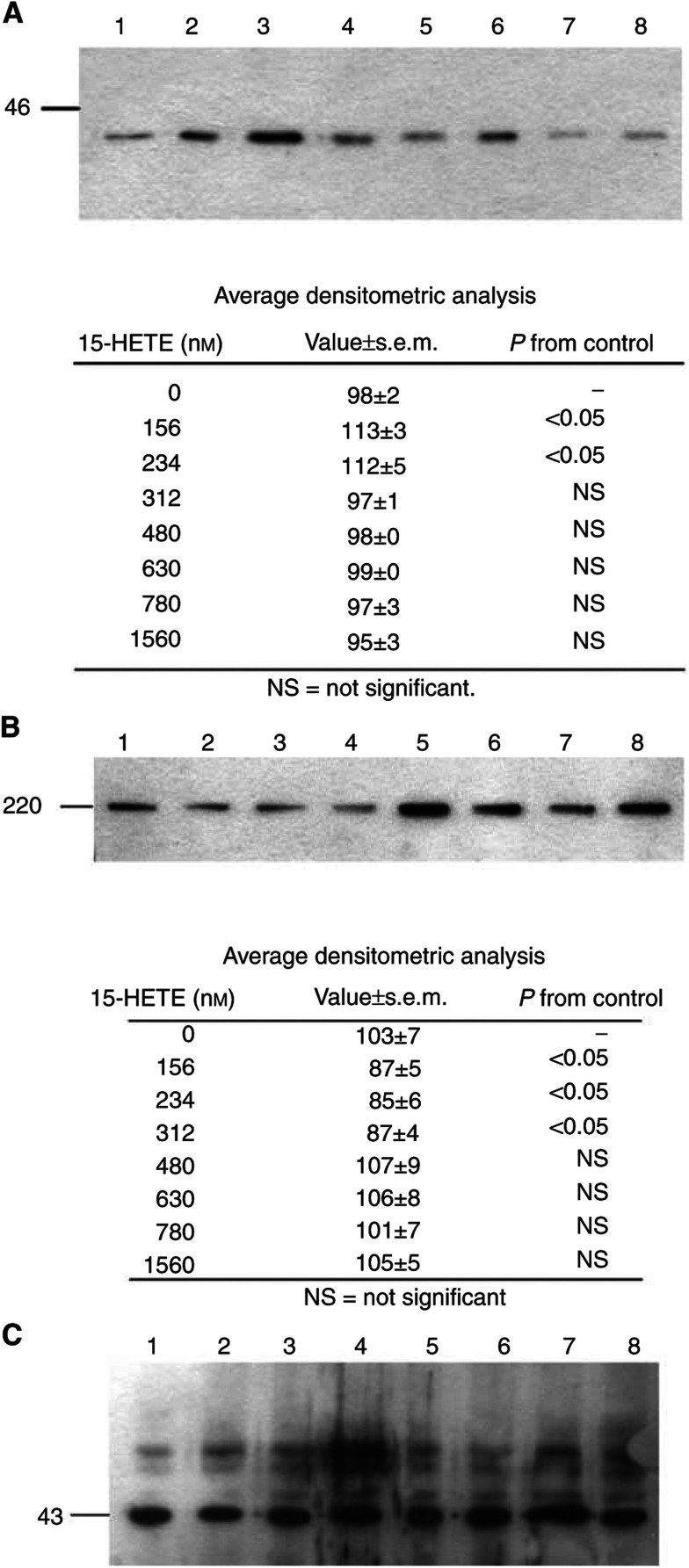
Western blots of soluble extracts of C_2_C_12_ myotubes treated with 0 (lane 1), 156 nm (lane 2), 234 nm (lane 3), 312 nm (lane 4), 480 nm (lane 5), 630 nm (lane 6), 780 nm (lane 7) and 1560 nM 15(*S*)-HETE, (lane 8) for 24 h on expression of p42 subunit of 19S regulatory subunit (**A**), myosin (**B**) and actin control (**C**). Blots were developed with the respective antibodies as described in Materials and methods, and are representative of three separate determinations on different samples. Average densitometric data are presented under the blots.

To determine whether increased mRNA expression for proteasome subunits C2 and C5 was reflected in higher protein levels of other subunits, cellular supernatants of C_2_C_12_ myotubes treated with 15(*S*)-HETE for 24 h were immunoblotted for p42, an ATPase subunit of the 19S regulator that promotes ATP-dependent association of the 20S proteasome with the 19S regulator to form the 26S proteasome (Tanahashi *et al*, 1999) (Figure 7A

). Maximal expression was seen at concentrations of 15(*S*)-HETE between 156 and 312 nM, which is in the range where an increased proteasome mRNA expression was seen (Figure 3). Activation of proteasome expression was associated with a decrease in myosin expression in the same concentration range (78–312 nM) (Figure 7B). These results suggest that 15(*S*)-HETE leads to an increased expression of the ubiquitin–proteasome pathway, with an increase in overall protein degradation and depletion of myofibrillar pro teins.

**Figure 8:**
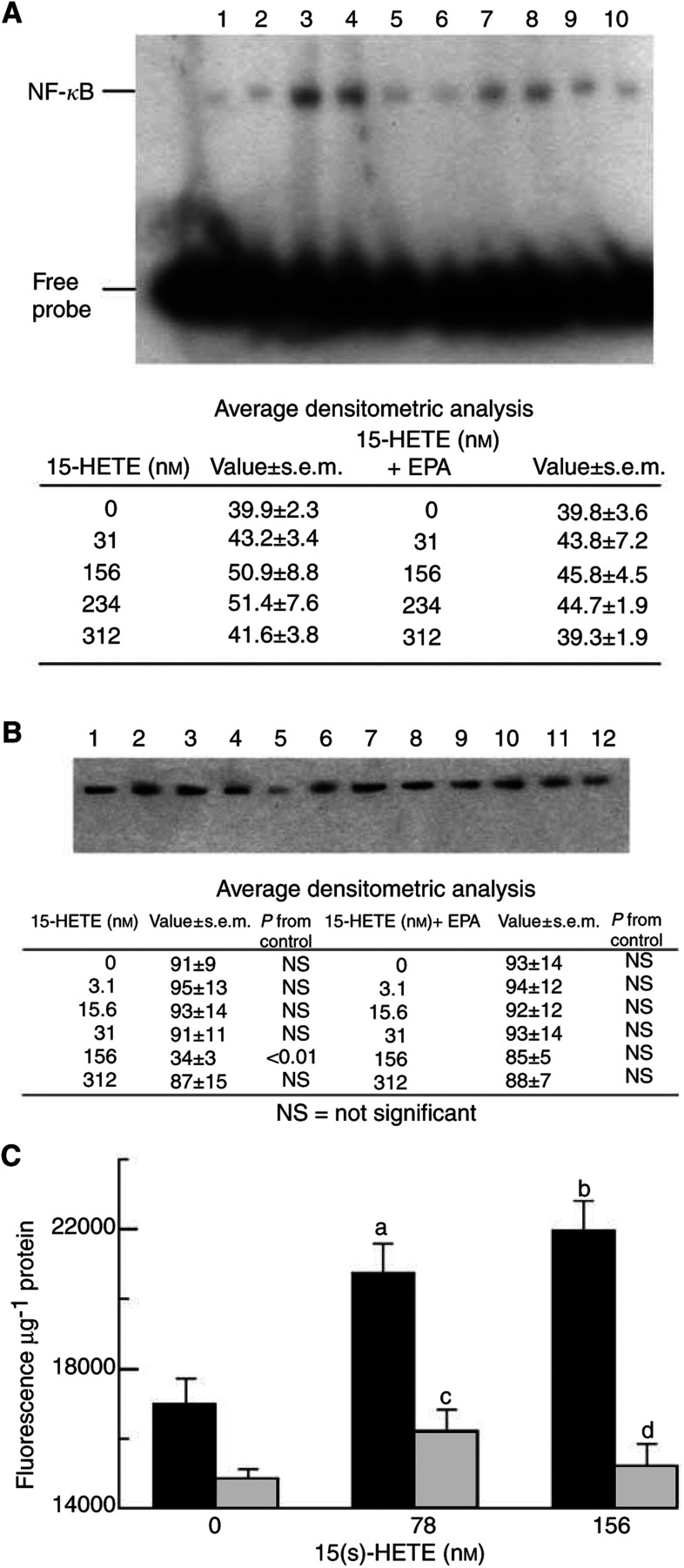
(**A**) Activation of NF-*κ*B binding to DNA as demonstrated by EMSA. Nuclear extracts of C_2_C_12_ myotubes treated with 0 nm (lane 1), 31 nm (lane 2), 156 nm (lane 3), 234 nm (lane 4) or 312 nM 15(*S*)-HETE (lane 5) for 30 min were compared with cells treated with 0 nm (lane 6), 31 nm (lane 7), 156 nm (lane 8), 234 nm (lane 9) or 156 nM 15(*S*)-HETE (lane 10) pretreated with 50 *μ*M EPA for 2 h. The gel shown is a representative example of three separate determinations and the average densitometric data are presented below the blot. (**B**) Western blot of I-*κ*B*α* levels in cytoplasmic extracts of myotubes treated with 0 nm (lane 1), 3.1 nm (lane 2), 15.6 nm (lane 3), 31 nm (lane 4), 156 nm (lane 5) and 312 nM 15(*S*)-HETE (lane 6) for 30 min or after pretreatment for 2 h with EPA (50 *μ*M) and treated with 0 nm (lane 7), 3.1 nm (lane 8), 15.6 nm (lane 9), 31 nm (lane 10), 156 nm (lane 11) and 312 nM 15(*S*)-HETE (lane 12). Detection was by rabbit polyclonal antisera to residues 1–317 of human I-*κ*B*α*. The blot shown is representative of three separate determinations and the average densitometric data are provided below the blot. (**C**) The effect of the NF-*κ*B inhibitor peptide SN50 on 15(*S*)-HETE induced upregulation of the ‘chymotrypsin-like’ activity of the proteasome. Myotubes were treated with the cell-permeable NF-*κ*B inhibitor peptide, SN50 (18 *μ*M) for 20 min prior to the addition of 15(*S*)-HETE. The activity shown represents the lactacystin (10 *μ*M) suppressible proteolytic activity. Differences are indicated as (a) *P*<0.05 and (b) *P*<0.01 from those in the absence of 15(*S*)-HETE and (c) *P*<0.01 and (d) *P*<0.001 from those in the absence of SN50.

The NF-*κ*B transcriptional pathway was investigated as a possible mediator of the increased expression of the ubiquitin–proteasome pathway, since NF-*κ*B has been shown to be an essential mediator of TNF-*α*-induced protein catabolism in differentiated muscle cells (Li and Reid, 2000). As shown in Figure 8A

, 15(S)-HETE induced elevated levels of NF-*κ*B DNA binding activity in the myotube nucleus, after a 30 min incubation, with a maximal effect between 156 and 234 nM. Increased nuclear accumulation of NF-*κ*B was not seen after pretreatment with EPA. Depletion of I-*κ*B*α* from the cytosol was determined by immunoblotting of cytoplasmic extracts from the same cells in which NF-*κ*B translocation was measured. Rapid breakdown of I-*κ*B*α* was evident 30 min after treatment with 156 nM 15(*S*)-HETE (Figure 8B), and this was not seen in cultures pretreated with 50 *μ*M EPA, suggesting a mechanism by which EPA could downregulate the increased proteasome expression seen in the presence of 15(*S*)-HETE. The NF-*κ*B inhibitor peptide, SN50 (Lin *et al*, 1995) attenuated the increased chymotrypsin-like enzyme activity in the presence of 15(*S*)-HETE (Figure 8C), suggesting the NF-*κ*B may be involved in transcriptional activation induced by 15(*S*)-HETE.

## DISCUSSION

There are three main pathways for the intracellular catabolism of proteins in skeletal muscle; lysosomal proteases, a nonlysosomal calcium-activated process involving the cysteine proteases calpains I and II and the ubiquitin–proteasome proteolytic pathway. Of the three, the latter is considered to be most important (Lecker *et al*, 1999), since the former two processes contribute less than 15–20% of total protein breakdown and do not catabolise myofibrillar proteins (Lowell *et al*, 1986). In cancer cachexia, where there is extensive and specific loss of skeletal muscle, tissue levels of mRNA for ubiquitin, 20S proteasome *α* and *β* subunits and the ubiquitin-conjugating enzyme, E2_14k_, have been found to be increased in mouse (Lorite *et al*, 1998), rat (Temparis *et al*, 1994) and humans (Williams *et al*, 1999). Knowledge of the mechanism of this increased gene transcription is important for the design of therapeutic agents, because muscle wastage leads to weakness, fatigue and eventually death through impairment of respiratory muscle function (Windsor and Hill, 1998).

Previous studies on protein degradation, induced by PIF *in vitro*, suggested 15(*S*)-HETE as a possible intracellular signal responsible for the increased transcription of the genes of the ubiquitin–proteasome proteolytic pathway (Smith *et al*, 1999). This suggestion is supported by recent observations (Whitehouse *et al*, 2001) showing that EPA, an inhibitor of 15(*S*)-HETE, produced in response to PIF, attenuated the development of muscle catabolism in the MAC16 murine cachexia model by downregulation of the increased expression of proteasome *α*-subunits and chymotrypsin-like enzyme activity. In addition, we have recently found (Whitehouse and Tisdale, 2001) that both EPA and a 12/15-lipoxygenase inhibitor, CV-6504, were also effective in attenuating protein degradation in acute starvation mediated through the ubiquitin–proteasome pathway. This suggests that in both models of muscle wasting the same intracellular-mediator is involved, possibly 15(*S*)-HETE. The aim of the present study has been to determine whether 15(*S*)-HETE alone is capable of upregulation of the key enzymes of the ubiquitin–proteasome pathway using C_2_C_12_ myotubes.

Using this *in vitro* system, 15(*S*)-HETE has been shown to induce total protein degradation at concentrations between 78 and 312 nM, with a bell-shaped dose–response curve similar to that previously reported in C_2_C_12_ myoblasts (Smith *et al*, 1999), although the concentration to produce a maximal effect was higher in myotubes. Since the effect of PIF on protein catabolism in myotubes has been shown to be mediated through an upregulation of the ubiquitin–proteasome proteolytic pathway (Gomes-Marcondes *et al*, 2002), if 15(*S*)-HETE was acting as an intracellular transducer of PIF action then the effect on protein degradation would also be mediated by this pathway. Accordingly 15(*S*)-HETE was shown to increase the ‘chymotrypsin-like’ enzyme activity of the *β* subunits of the proteasome, suggested to be the rate-determining step in protein catabolism (Chen and Hochstrasser, 1996; Rock *et al*, 1994), in the same concentration range as that stimulating total protein degradation. Further confirmation that 15(*S*)-HETE stimulated expression of the rate-limiting components of the ubiquitin–proteasome pathway was provided by an increased mRNA level for proteasome subunits C2 and C5, as well as E2_14k_, and increased protein levels of proteasome subunit, p42, over the same concentration range as that inducing total protein degradation. Previous studies have reported increased proteasome subunits (Temparis *et al*, 1994; Lorite *et al*, 1998) MSS1 (Combaret *et al*, 1999) and E2_14k_ with increased muscle catabolism in cancer cachexia, suggesting that they play important roles in protein degradation. Indeed, cellular levels of the myofibrilar protein, myosin, were reduced in myotubes treated with 15(*S*)-HETE over the same concentration range as that inducing the regulatory components of the ubiquitin–proteasome proteolytic pathway.

We have suggested (Whitehouse *et al*, 2001) that EPA attenuates protein degradation and the increased expression of the key regulatory components of the ubiquitin–proteasome pathway as a result of the inhibition of 15(*S*)-HETE production through the PIF-induced activation of phospholipase A_2_ and arachidonate release (Smith *et al*, 1999). It was therefore surprising that EPA was able to attenuate protein degradation and the increase in proteasome expression induced by 15(*S*)-HETE. This suggests that EPA also affects a downstream pathway involved in induction of proteasome expression by 15(*S*)-HETE, possibly a nuclear transcription factor, since Ross *et al* (1999) have shown that in the pancreatic cancer cell line, MiaPaCa 2, exposed to EPA for 2 h prior to a pulse of TNF-*α*, I-*κ*B*α* was preserved, suggesting an effect of EPA in stabilising the NF-*κ*B complex in the cytoplasm.

Watchorn *et al* (2001) have shown PIF to activate both the transcription factors NF-*κ*B and STAT3 in hepatic cells. Li *et al* (1998) showed that TNF-*α* stimulated protein loss in C_2_C_12_ myotubes involved the ubiquitin–proteasome proteolytic pathway and was accompanied by nuclear translocation of NF-*κ*B to its targeted DNA sequence by stimulating phosphorylation, ubiquitin conjugation and proteolysis of I-*κ*B*α*. Total protein content and myosin heavy chain levels in C_2_C_12_ myotubes were found to be unaltered in cells transfected with viral plasmid constructs that overexpress mutant I-*κ*B*α* proteins that are insensitive to degradation by the ubiquitin–proteasome pathway (Li and Reid, 2000). These data suggest that NF-*κ*B is an essential mediator of TNF-*α*-induced protein catabolism in differentiated muscle cells. However, Du *et al* (2000) found that NF-*κ*B is a repressor of C3 proteasome subunit transcription in muscle cells and that glucocorticoids stimulate C3 proteasome subunit expression by opposing this suppressor action. This mechanism is surprising, since NF-*κ*B normally acts as a transcriptional activator, and the present study provides some evidence that NF-*κ*B may be involved in the increase in transcription of the regulatory components of the ubiquitin–proteasome pathway induced by 15(*S*)-HETE. Thus, 15(*S*)-HETE induced elevated levels of NF-*κ*B binding activity in the nucleus of C_2_C_12_ myotubes with a corresponding decrease in cytosolic I-*κ*B*α*. The effect was not seen in myotubes pretreated with EPA, providing a role for EPA in the attenuation of downstream signalling induced by 15(*S*)-HETE. The concentrations of 15(*S*)-HETE affecting the NF-*κ*B/I-*κ*B*α* system were the same as those increasing protein degradation and stimulating increased expression of the key regulatory components of the ubiquitin–proteasome pathway. Moreover, the NF-*κ*B inhibitor peptide SN50 (Lin *et al*, 1995) attenuated the increased proteasome chymotryptic activity in the presence of 15(*S*)-HETE. These results suggests that 15(*S*)-HETE may induce gene expression through the transcription factor NF-*κ*B.

The mechanism by which 15(*S*)-HETE could activate NF-*κ*B is not known. One possibility is that as 15(*S*)-HETE is oxidised it could result in the generation of reactive oxygen species, which might regulate the redox sensitive NF-*κ*B, possibly through oxidation of constituent proteins, which could augment DNA-binding activity or promote the release from, or degradation of I-*κ*B. Mild oxidative stress has been shown to induce 20S proteasome subunits and E2_14k_ in murine myotubes (Gomes-Marcondes and Tisdale, 2002). However, it is not clear why 5- and 12-HETE would not have the same properties as 15-HETE if this were the mechanism. Further studies are required to determine the role of transcription factors and cellular signalling pathways in the action of 15(*S*)-HETE.

## References

[bib1] Adegoke OA, Bedard N, Roest HP, Wing SS (2002) Ubiquitin-conjugating enzyme E214k/HR6B is dispensable for increased protein catabolism in muscle of fasted mice. Am J Physiol 283: E482–E489 10.1152/ajpendo.00097.2002 12169441

[bib2] Bodine SC, Latres E, Baumhueter S, Lai VK, Nunez L, Clarke BA, Poueymirou WT, Panaro FJ, Na E, Dharmarajan K, Pan ZQ, Valenzuela DM, DeChiara TM, Stitt TN, Yancopoulos GD, Glass DJ (2001) Identification of ubiquitin ligases required for skeletal muscle atrophy. Science 294: 1704–1708 11679633 10.1126/science.1065874

[bib3] Chen P, Hochstrasser M (1996) Autocatalytic subunit processing couples active site formation in the 20S proteasome to completion of assembly. Cell 86: 961–972 8808631 10.1016/s0092-8674(00)80171-3

[bib5] Ciechanover A, Orian A, Schwartz AL (2000) The ubiquitin-mediated proteolytic pathway: mode of action and clinical implications. J Cell Biochem 34: 40–51 10.1002/(sici)1097-4644(2000)77:34+<40::aid-jcb9>3.0.co;2-6 10762014

[bib6] Combaret L, Ralliere C, Taillandier D, Tanaka K, Attaix D (1999) Manipulation of the ubiquitin–proteasome pathway in cachexia: pentoxifylline suppresses the activation of 20S and 26S proteasomes in muscles from tumor-bearing rats. Mol Biol Rep 26: 95–101 10363654 10.1023/a:1006955832323

[bib9] DeBlaauw I, Deutz NEP, von Meyenfeldt MF (1997) Metabolic changes in cancer cachexia. Clin Nutr 16: 169–176 16844595 10.1016/s0261-5614(97)80002-7

[bib10] Du J, Mitch WE, Wang X, Price SR (2000) Glucocorticoids induce proteasome C3 subunit expression in L6 muscle cells by opposing the suppression of transcription by NF-*κ*B. J Biol Chem 275: 19661–19666 10867022 10.1074/jbc.M907258199

[bib11] Fentiny G, Schreiber SL (1998) Lactacystin, proteasome function and cell fate. J Biol Chem 273: 8545–8548 9535824 10.1074/jbc.273.15.8545

[bib12] Gomes MD, Lecker SH, Jagoe RT, Navon A, Goldberg A (2001) Atrogin-1, a muscle-specific F-box protein highly expressed during muscle atrophy. Proc Natl Acad Sci USA 98: 14440–14445 11717410 10.1073/pnas.251541198 PMC64700

[bib13] Gomes-Marcondes MCC, Smith HJ, Cooper JC, Tisdale MJ (2002) Development of an *in vitro* model system to investigate the mechanism of muscle protein catabolism induced by proteolysis-inducing factor. Br J Cancer 86: 1628–1633 12085214 10.1038/sj.bjc.6600236 PMC2746596

[bib14] Gomes-Marcondes MCC, Tisdale MJ (2002) Induction of protein catabolism and the ubiquitin–proteasome pathway by mild oxidative stress. Cancer Lett 180: 69–74 11911972 10.1016/s0304-3835(02)00006-x

[bib15] Lecker SH, Solomon V, Mitch WE, Goldberg AL (1999) Muscle protein breakdown and the critical role of the ubiquitin–proteasome pathway in normal and disease states. J Nutr 129: 227S–237S 9915905 10.1093/jn/129.1.227S

[bib16] Li Y-P, Reid MB (2000) NF-*κ*B mediates the protein loss induced by TNF-*α* in differentiated skeletal muscle myotubes. Am J Physiol 279: R1165–R1170 10.1152/ajpregu.2000.279.4.R1165 11003979

[bib17] Li Y-P, Schwartz RJ, Waddell ID, Holloway BR, Reid MB (1998) Skeletal muscle myocytes undergo protein loss and reactive oxygen-mediated NF-*K*B activation in response to tumor necrosis factor *α*. FASEB J 12: 871–880 9657527 10.1096/fasebj.12.10.971

[bib18] Lin Y-Z, Yao SY, Veach RA, Torgerson TR, Hawiger J (1995) Inhibition of nuclear translocation of transcription factor NF-*κ*B by a synthetic peptide containing a cell membrane-permeable motif and nuclear localization sequence. J Biol Chem 270: 14255–14258 7782278 10.1074/jbc.270.24.14255

[bib19] Lorite MJ, Cariuk P, Tisdale MJ (1997) Induction of muscle protein degradation by a tumour factor. Br J Cancer 76: 1035–1040 9376263 10.1038/bjc.1997.504 PMC2228106

[bib20] Lorite MJ, Thompson MG, Drake JL, Carling G, Tisdale MJ (1998) Mechanism of muscle protein degradation induced by a cancer cachectic factor. Br J Cancer 76: 850–856 10.1038/bjc.1998.592 PMC2063122 9764574

[bib21] Lorite MJ, Smith HJ, Arnold JA, Morris A, Thompson MG, Tisdale MJ (2001) Activation of ATP-ubiquitin-dependent proteolysis in skeletal muscle *in vivo* and murine myoblasts *in vitro* by a proteolysis-inducing factor (PIF). Br J Cancer 85: 297–302 11461093 10.1054/bjoc.2001.1879 PMC2364050

[bib22] Lowell BB, Ruderman NB, Goodman MN (1986) Evidence that lysosomes are not involved in the degradation of myofibrillar proteins in rat skeletal muscle. Biochem J 234: 237–240 3707546 10.1042/bj2340237 PMC1146553

[bib23] Mitch WE, Bailey JL, Wang X, Jurkovitz C, Newby D, Price SR (1999) Evaluation of signals activating ubiquitin–proteasome proteolysis in a model of muscle wasting. Am J Physiol 276: C1132–C1138 10329962 10.1152/ajpcell.1999.276.5.C1132

[bib24] Orino E, Tanaka K, Tamura T, Sone S, Ogura T, Ichihara A (1991) ATP-dependent reversible association of proteasomes with multiple protein components to form 26S complexes that degrade ubiquitinated proteins in human HL-60 cells. FEBS Lett 284: 206–210 1647982 10.1016/0014-5793(91)80686-w

[bib25] Price SR, England BK, Bailey JL, Van Vreede K, Mitch WE (1994) Acidosis and glucocorticoids concomitantly increase ubiquitin and proteasome subunit mRNAs in rat muscle. Am J Physiol 267: C955–C960 7943291 10.1152/ajpcell.1994.267.4.C955

[bib26] Ralliere C, Tauveron I, Taillandier D, Guy L, Boiteux JP, Giraud B, Attaix D, Thieblot P (1997) Glucocorticoids do not regulate the expression of proteolytic genes in skeletal muscle from Cushing's syndrome patients. J Clin Endocrinol Metab 82: 3161–3164 9284762 10.1210/jcem.82.9.4221

[bib27] Rock KL, Gramm C, Rothstein L, Clark K, Stein R, Dick L, Hwang D, Goldberg AL (1994) Inhibitors of the proteasome block the degradation of most cell proteins and the generation of peptides presented on MHC class 1 molecules. Cell 78: 761–771 8087844 10.1016/s0092-8674(94)90462-6

[bib28] Ross JA, Moses AG, Fearon KCH (1999) The anti-catabolic effects of n-3 fatty acids. Curr Opin Clin Nutri Metab Care 2: 219–226 10.1097/00075197-199905000-00005 10456251

[bib29] Smith HJ, Lorite MJ, Tisdale MJ (1999) Effect of a cancer cachectic factor on protein synthesis/degradation in murine C_2_C_12_ myoblasts: modulation by eicosapentaenoic acid. Cancer Res 59: 5507–5513 10554027

[bib30] Tanahashi N, Kawahara H, Murakami Y, Tanaka K (1999) The proteasome-dependent proteolytic system. Mol Biol Rep 26: 3–9 10363639 10.1023/a:1006909522731

[bib31] Taylor SL, Higgins AP, Rudland PS, Winstanley JHR, Barraclough R (1998) Cytoplasmic staining of c-erb B-2 is not associated with the presence of detectable c-erb B-2 mRNA in breast cancer specimens. Int J Cancer 76: 459–463 9590117 10.1002/(sici)1097-0215(19980518)76:4<459::aid-ijc2>3.0.co;2-q

[bib32] Temparis S, Asensi M, Taillandier D, Aurousseau E, Larbaud D, Obled A, Becket D, Ferrara M, Estrela JM, Attaix D (1994) Increased ATP-ubiquitin-dependent proteolysis in skeletal muscles of tumor-bearing rats. Cancer Res 54: 5568–5573 7923198

[bib33] Tiao G, Fagin J, Roegner V, Lieberman M, Wang JJ, Fischer JE, Hasselgren P-O (1996) Energy-ubiquitin-dependent muscle proteolysis during sepsis in rats is regulated by glucocorticoids. J Clin Invest 97: 339–348 8567953 10.1172/JCI118421 PMC507023

[bib34] Todorov P, Cariuk P, McDevitt T, Coles B, Fearon K, Tisdale M (1996) Characterization of a cancer cachectic factor. Nature 379: 739–742 8602222 10.1038/379739a0

[bib35] Watchorn TM, Waddell ID, Dowidar N, Ross JA (2001) Proteolysis-inducing factor regulates hepatic gene expression via the transcription factors NF-*κ*B and STAT3. FASEB J 15: 562–564 11259367 10.1096/fj.00-0534fje

[bib36] Wang AM, Doyle MV, Marks DF (1989) Quantitation of mRNA by the polymerase chain reaction. Proc Natl Acad Sci USA 86: 9717–9721 2481313 10.1073/pnas.86.24.9717 PMC298572

[bib37] Whitehouse AS, Tisdale MJ (2001) Downregulation of ubiquitin-dependent proteolysis by eicosapentaenoic acid in acute starvation. Biochem Biophys Res Commun 285: 598–602 11453634 10.1006/bbrc.2001.5209

[bib87] Whitehouse AS, Smith HJ, Drake JL, Tisdale MJ (2001) Mechanism of attenuation of skeletal muscle protein catabolism in cancer cachexia by eicosapentaenoic acid. Cancer Res 61: 3604–3609 11325828

[bib39] Williams A, Sun X, Fischer JE, Hasselgren P-O (1999) The expression of genes in the ubiquitin–proteasome proteolytic pathway is increased in skeletal muscle from patients with cancer. Surgery 126: 744–750 10520924

[bib40] Windsor JA, Hill GL (1988) Risk factors for postoperative pneumonia. The importance of protein depletion. Ann Surg 208: 209–217 3401064 10.1097/00000658-198808000-00013 PMC1493609

[bib41] Wing SS, Goldberg AL (1993) Glucocorticoids activate the ATP-ubiquitin-dependent proteolytic system in skeletal muscle during fasting. Am J Physiol 264: E668–E676 7682781 10.1152/ajpendo.1993.264.4.E668

[bib42] Wing SS, Banville D (1994) 14-kDa ubiquitin-conjugating enzyme. Structure of the rat gene and regulation upon fasting and by insulin. Am J Physiol 267: E39–E48 8048511 10.1152/ajpendo.1994.267.1.E39

